# New Insights Into Tissue Culture Plant-Regeneration Mechanisms

**DOI:** 10.3389/fpls.2022.926752

**Published:** 2022-06-30

**Authors:** Yun Long, Yun Yang, Guangtang Pan, Yaou Shen

**Affiliations:** ^1^Key Laboratory of Southwest China Wildlife Resources Conservation (Ministry of Education), College of Life Science, China West Normal University, Nanchong, China; ^2^Nanchong Academy of Agricultural Sciences, Nanchong, China; ^3^Key Laboratory of Biology and Genetic Improvement of Maize in Southwest Region, Maize Research Institute, Sichuan Agricultural University, Chengdu, China

**Keywords:** somatic embryogenesis, *de novo* organogenesis, environmental factors, molecular mechanisms, plant regeneration

## Abstract

Plant regeneration occurs when plants repair or replace damaged structures based on the totipotency and pluripotency of their cells. Tissue culture is one of the most widely used regenerative technologies. Recently, a series of breakthroughs were made in the study of plant regeneration. This review summarizes two regenerative pathways in tissue culture: somatic embryogenesis and *de novo* organogenesis. Furthermore, we review the environmental factors influencing plant regeneration from explant sources, basal culture medium, plant growth regulators, and light/dark treatment. Additionally, we analyse the molecular mechanisms underlying two pathways. This knowledge will promote an understanding of the fundamental principles of plant regeneration from precursor cells and lay a solid foundation for applying plant micropropagation and genetic modification.

## Introduction

An entire plant can be regenerated from an adult tissue or organ, a mass of unorganized calli, or even a single cell in a process referred to as plant regeneration. Plant regeneration refers to the physiological renewal, repair, or replacement of tissue in plants (Ikeuchi et al., 2016). The totipotency or pluripotency of plant cells underlies the ability of plants to regenerate, reflecting the high plasticity of cell fate. Totipotency refers to the ability of a cell to differentiate into a complete individual, whereas pluripotency involves the differentiation of a specific group of tissues or organs from a cell (Verdeil et al., 2007). The concept of tissue culture was proposed as early as a century ago and envisaged the regeneration of whole plants from somatic cells *in vitro* (Haberlandt, 1902). The tissue culture system has matured since the historical discovery that different concentration ratios of auxin and cytokinin (CK) are critical to regenerating adventitious roots and shoots (Skoog and Miller, 1957). Steward et al. (1958) successfully regenerated new somatic embryos and subsequently developed roots and shoots by using isolated phloem cells from carrot roots, which confirmed the totipotency of plant cells. Since then, tissue culture technology based on regenerative ability has been extensively used in various fields, including basic research, micropropagation, and transgenic breeding.

The ability of plant regeneration is affected by multiple factors, including use of a plant growth regulator (PGR; Çabuk and Özgen, 2016; Gerdakaneh et al., 2020), the composition of basic medium (Sundararajan et al., 2017; Chimdessa, 2020), and explant type (Dhar and Joshi, 2005; Minutolo et al., 2020). Importantly, plant tissue culture presents strong species dependence and genotype specificity. Some plants, such as tobacco (*Nicotiana tabacum*), *Arabidopsis thaliana*, and rice (*Oryza sativa*), can be easily regenerated *in vitro*, whereas other plants, such as soybean (*Glycine Max*), wheat (*Triticum aestivum*), and maize (*Zea mays*), are more difficult to regenerate. Moreover, *Japonica* varieties show a higher capacity for callus formation than *Indica* varieties in rice (Abe and Futsuhara, 1986). The tissue culture capacities of hybrid lines are higher than those of inbred lines in maize (Duncan et al., 1985). Clarifying the regulatory network and genetic control of plant-regeneration ability in tissue culture is helpful to improving plant-regeneration rates and genetic transformation efficiency.

Therefore, this review discusses two pathways of plant regeneration in tissue culture: somatic embryogenesis and *de novo* organogenesis. We then describe how environmental factors affect plant regeneration from the aspects of explant sources, basal culture medium, PGRs, and light/dark treatment. Importantly, we describe the molecular mechanisms that regulate somatic embryogenesis from three levels: transcription factors, hormone signalling, and epigenetic regulation. Furthermore, we elaborate on the molecular mechanisms underlying pluripotent callus formation, *de novo* root organogenesis, and *de novo* shoot organogenesis. This review provides insight into how plants regenerate from explants and important cues for plant micropropagation and genetic modification.

## Pathways of Plant Regeneration in Tissue Culture

Regeneration pathways in seed plants can be divided into tissue repair, somatic embryogenesis, and *de novo* organogenesis. The first pathway concerns how young plant tissues, such as root or leaf tips, repair injured parts and is often used in plant-cutting propagation techniques (Xu and Huang, 2014). In tissue culture, plants are regenerated mainly by somatic embryogenesis and *de novo* organogenesis (Hill and Schaller, 2013).

### Somatic Embryogenesis

In somatic embryogenesis, plant somatic cells undergo dedifferentiation into embryonic stem cells and then by way of embryonic development form complete plants, signifying that plant cells are totipotent by virtue of the embryogenic callus (Zimmerman, 1993; Verdeil et al., 2007). Somatic embryogenesis leads to an exchange in cell fate from a somatic cell back into an embryonic stem cell. Dedifferentiation through this pathway is usually accomplished under stress conditions, hormonal induction (e.g., auxin), or gene expression modification (Jiménez and Thomas, 2006; Fehér, 2015; Horstman et al., 2017). Somatic embryos can be directly induced from individual somatic cells or indirectly generated from embryonic callus (Yang and Zhang, 2010; Horstman et al., 2017).

**Figure 1:**
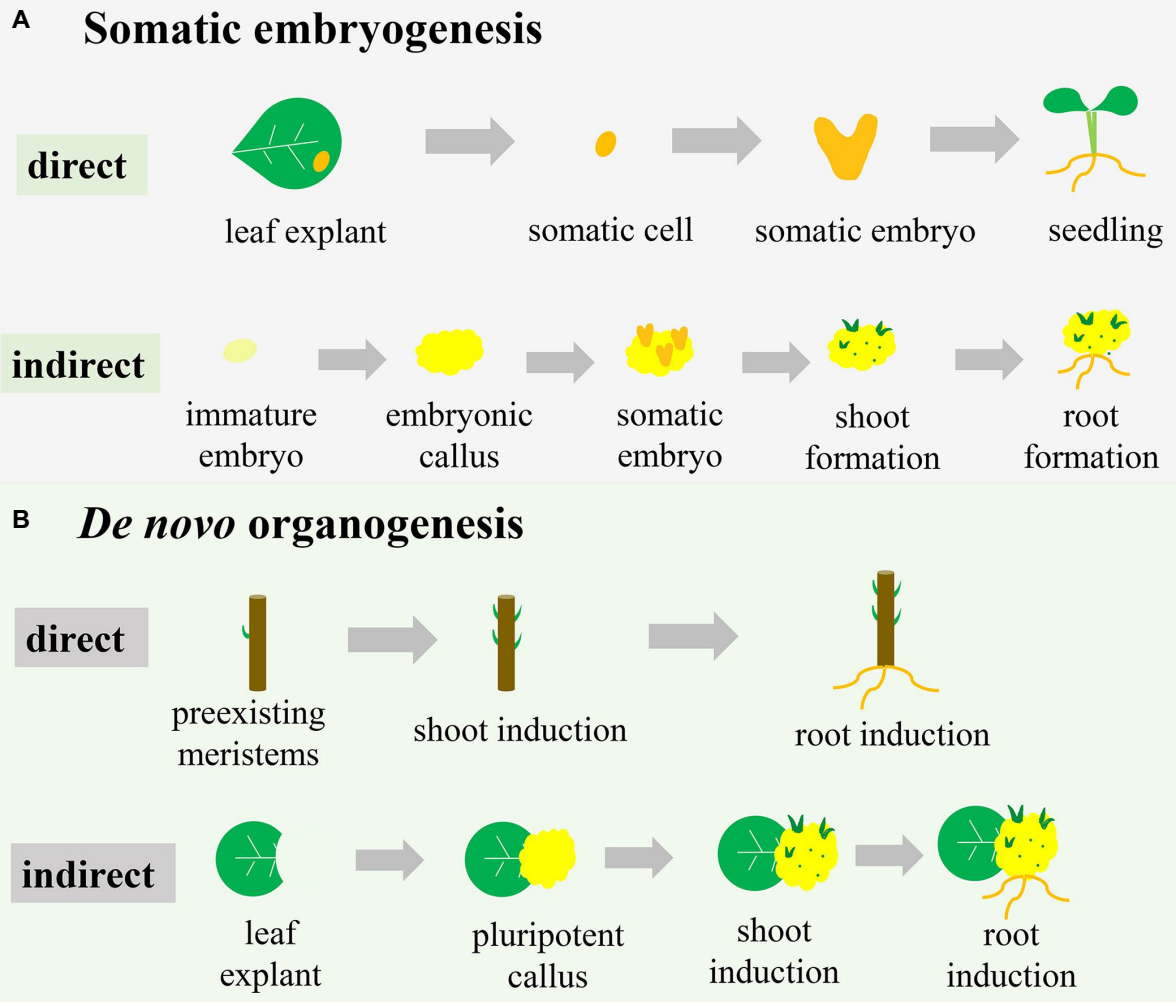
Different pathways of plant regeneration. **(A)** Somatic embryogenesis. In the direct pathway, the somatic cell originated from explants (e.g., a leaf) is induced to form the somatic embryo, which subsequently drives the development of the whole plant. In the indirect pathway, the explant (e.g., an immature embryo) is induced to initiate the embryonic callus, on which somatic embryos are formed to subsequently develop shoots and roots. **(B)** *De novo* organogenesis. In the direct pathway, shoots and roots are induced directly on the stem with pre-existing meristems. In the indirect pathway, pluripotent callus is produced around the wound in a leaf explant, with formation of shoots and roots subsequently induced.

Indirect somatic embryogenesis is the most common pathway, especially in crop plants, and starts with the embryonic callus (an unorganized cell mass; Figure 1A). Embryonic callus induction is followed by the formation of proembryonic masses on the surface or within the callus mass, from which single cells or cell clusters develop into somatic embryos (Toonen et al., 1994). Under appropriate conditions, somatic embryos can develop into shoots and roots (Figure 1A). In the case of maize (Rakshit et al., 2010), embryonic callus can be induced to form from explants, such as immature embryos and shoot tips, in a callus-inducing medium containing a high level of auxin and a low level of CK. When transferred to a shoot-inducing medium (SIM) containing a high level of CK and a low level of auxin, embryonic callus differentiates into shoots. For root regeneration, root-inducing medium containing some auxin without CK is required for incubating embryonic callus.

Unlike the formal pathway, direct somatic embryogenesis lacks the callus phase and is less well defined (Figure 1A). In this system, the explant exhibits a more regular compact cell division and is less prolific (Horstman et al., 2017). The individual somatic cell in one or more cell layers divides and bulges under appropriate conditions to develop into a morphologically recognizable new embryo capable of developing into a whole plant (Fitch and Manshardt, 1990; Xu and Huang, 2014; Figure 1A). For example, constitutive expression of the morphogenic transcription factors *BABY BOOM* (*BBM*) and *WUSCHEL (WUS)2* in maize resulted in rapid formation of abundant somatic embryos on the scutella (Lowe et al., 2018). These somatic embryos could then be directly germinated into plants without the callus phase.

Direct and indirect somatic embryogenesis pathways can occur in the same explant, but the periods of obtaining regenerated plants differ (Zhang et al., 2021). Compared with the direct somatic embryogenesis pathway, the indirect pathway has a longer period to regenerate plants due to the callus-induction process. Therefore, the indirect somatic embryogenesis pathway is frequently associated with somaclonal variation (Miguel and Marum, 2011; Bahmankar et al., 2017). However, the indirect somatic embryogenesis pathway results in more regenerated plantlets than the direct pathway due to the plentiful proliferation of callus (Gaj, 2011). Therefore, if the goal is rapid regeneration of plants, the direct pathway is more efficient than the indirect pathway. However, for species in which explants are difficult to obtain or situations where many regenerated plants are desired, the indirect pathway is the better choice.

### *De novo* Organogenesis

*De novo* organogenesis refers to the regenerative process that does not use a somatic embryo but rather the differentiation of the meristematic centre, reflecting the pluripotency of plant cells (Lardon and Geelen, 2020). Plant-regenerative mechanisms, such as *de novo* organogenesis, result in regenerating adventitious roots and/or adventitious shoots *in vitro* or from injured plant organs, with this frequently occurring in nature (Duclercq et al., 2011). The regeneration process of adventitious shoots and roots is referred to as *de novo* shoot organogenesis and *de novo* root organogenesis. Like somatic embryogenesis, *de novo* organogenesis can also be categorized as either a direct or indirect regeneration pathway (Figure 1B). As with somatic embryogenesis, shoots or roots are directly induced from pre-existing meristems or injured organs under advisable conditions (Sang et al., 2018; Figure 1B). Cutting-propagation technology is based on the direct *de novo* organogenesis used to regenerate organs. During indirect *de novo* organogenesis, cells undergo dedifferentiation and plant growth regulators stimulate cell division (Sugimoto and Meyerowitz, 2013), after which additional dedifferentiated cells are induced with further culture time and ultimately generate pluripotent callus (Figure 1B). When all conditions are met, the pluripotent callus undergoes physiological and biochemical changes, resulting in different cell-division positions and directions (Wang et al., 2011; de Almeida et al., 2015). *De novo* shoot organogenesis and *de novo* root organogenesis are initiated using different combinations of auxin and CK (Street and Henshaw, 1966).

The essential difference between *de novo* organogenesis and somatic embryogenesis is the absence of somatic embryo formation, whereas both pathways include direct and indirect methods of regeneration (Figure 1). The callus is formed in the two indirect methods, but the characteristics of the callus differ. Somatic embryogenesis leads to embryogenic callus with totipotency and subsequent development into a somatic embryo, whereas *de novo* organogenesis induces non-embryogenic callus with pluripotency (Yumbla-Orbes et al., 2017; Shin and Seo, 2018; Fehér, 2019). Moreover, indirect *de novo* organogenesis can result in genetic instability and somaclonal variance similar to somatic embryogenesis (Vitamvas et al., 2019). Organ production directly from explants is a time-saving method but unsuitable for transgenic research due to the production of chimeric shoots containing both transformed and untransformed cells (Firoozabady and Moy, 2004). Many studies have reported the induction of embryonic tissue from immature seeds or embryos of cereal crops, suggesting that somatic embryogenesis is restricted to a certain time of year (Malik et al., 2004; Jones et al., 2019). However, the material used for organogenesis is multiplicative and seasonally flexible. Additionally, for some organs or tissues that are easy to induce *de novo* organogenesis, it might be difficult to develop somatic embryos. Therefore, two pathways are occasionally combined to enhance the frequency of plantlet regeneration in a giving species for commercial marketplace or scientific research (Abe and Futsuhara, 1986).

## Environmental Factors Affecting Plant Regeneration

### Explant Sources

Although all plant cells have the totipotential to regenerate entire plants, the ease in expression of that capacity varies in plant species and varieties, even in cells of the same plant (Table 1). For example, only a part of maize stock is capable of plant regeneration in tissue culture. These include a few self-inbred lines, F_1_ hybrids, and open-pollinated hybrids (Hibberd, 1984; Hodges et al., 2012). A previous study tested 101 maize self-inbred lines to examine the ability of plant regeneration, finding that only 49% were able to regenerate whole plants, with 97% of the hybrids producing callus capable of plant regeneration having at least one regenerable parent (Duncan et al., 1985). Another study evaluated a total of 113 tropical maize inbreds for tissue culture response, revealing that only 42 had the ability of embryonic callus induction (Carvalho et al., 1997). Moreover, the tissue culture capacities of hybrid lines are higher than those of inbred lines, although until recently, it remained difficult to explain this fact. Furthermore, conditions favourable for plant regeneration in one cultivar can sometimes be inadequate to grow plants in another cultivar of the same species (Ali et al., 2007; Satish et al., 2016).

**Table 1:** Environmental factors and molecular mechanisms affecting plant regeneration in tissue culture.

	Classifications	Specific contents
Environmental factors	Explant sources	Genotype (13, 41, 44–47); Age (50, 52, 55, 58, 60)
	PGRs	Auxin (56, 57, 76-79); CK (4, 80); ABA (81); GA (82)
	Basal culture medium	MS (83); N6 (8); B5 (83); WPM (84); carbon source (86–88)
	Light/Dark treatment	Photoperiod (91–93); light intensity (94); light type (95, 96)
Molecular mechanisms	Transcription factors	SERK1/2(116–121); WIND1 (113, 165); WUS (151, 152, 166–168); WOX5 (143); WOX11/12(145, 146); LEC1/2(100–106); BBM (102, 107); ABI3 (97–102); FUS3 (97–103); AGL15 (103, 104); CLV3 (153, 166-168); STM (154, 166–168); CUC1/2(141, 162); PLT 1/2(31); PLT3/5/7(148); EIN3 (147); LBD16 (148)
	DNA methylation	MET1 (131); CMT3 (132,155); DRM1/2(132, 155)
	Histone modifications	PRC1/2(14, 102, 133–135); PKL (136, 137); HADCs (138); HATs (138, 157); HAC1 (157)
	Auxin Signalling	IAA30 (103, 104); YUCS (19, 105, 106, 158); TAA1 (19, 105, 106); PIN (126); ARF (127, 128); AUX (126)
	Cytokinin Signalling	Type-A ARRs (113, 115, 155); Type-B ARRs (113, 115, 151)
	Other factors	miR160 (129); miR165/166(130, 160); miR156 (159)

The age of explants is another factor that affects plant regeneration in tissue culture (Table 1). Although every living cell can regenerate entire plants, most studies use cells or tissues with active growth and vigorous physiological metabolism as explants (Hoque and Mansfield, 2004). Among the explants used in tissue culture, the most widely used are immature embryos, including in maize (Jones et al., 2019), rice (Rakshana et al., 2019), wheat (Kumar et al., 2017), barley (Hinchliffe and Harwood, 2019), and other important cereal crops. Immature inflorescences are also suitable explants for sorghum (Chou et al., 2020), wheat (Mahmood and Razzaq, 2017), and barley (Saeedpour et al., 2021). Moreover, immature cotyledons and hypocotyl segments excised from seedlings are often utilized for medicinal plants, such as *Pterocarpus marsupium* (Husain et al., 2010), *Cassia angustifolia* (Parveen and Shahzad, 2014) and *Santalum album* L. (Akhtar and Shahzad, 2019). Additionally, embryogenic callus was successfully induced from young leaves in wheat (Yu et al., 2012), sorghum (Wernicke et al., 1982), and rye (Haliloglu and Aydin, 2016), and other explants have also been reported, including root tips (Wang et al., 2021), shoot tips (Long et al., 2020), anthers (Han et al., 2021), and pollen (Cho and Zapata, 1988). Regardless of the explant, initial cell division begins at a young part near the cambium and vascular bundles. Explants in the juvenile-development phase are more regenerative and possess higher totipotency than those of adult explants (Lee et al., 2020). For example, a study investigating the frequency of embryonic callus induction among different ages of maize seedlings found a higher frequency of embryonic callus induction for seedlings that were between 2- and 6-cm long than for longer seedlings (Long et al., 2020). Moreover, reports indicate that differences in endogenous hormones and nutrients in various parts of explants may explain the differences in regenerative abilities (Bhaskaran and Smith, 1990; Saeedpour et al., 2021), with variations in endogenous hormones also affecting the demand for exogenous hormones in tissue culture.

### Plant Growth Regulators

Exogenous hormones, especially auxin, CK, and other PGRs, play an important role in plant somatic embryogenesis and *de novo* organogenesis (Jiménez, 2005; Schwarz and Beaty, 2018; Table 1). Plant regeneration *in vitro* depends on the addition of exogenous hormones and the response to these hormones during tissue culture (Bernula et al., 2020). Generally, the response of explants to PGRs comprises three stages: (1) cultured explant cells perceive plant hormone signalling to induce subsequent dedifferentiation; (2) due to the influence of plant hormone balance, the differentiation instructions for specific cells in plant tissue are given, laying the foundation for the subsequent differentiation of specific organs; and (3) plant morphogenesis occurs independent of exogenous hormones (Ye et al., 2012). Although somatic embryogenesis is initiated by exogenous auxin, its further occurrence does not require auxin. A possible reason is that exogenous auxin promotes the synthesis of endogenous auxin, with the resulting increases in endogenous auxin promoting regeneration (Michalczuk et al., 1992; Nic-Can and Loyola-Vargas, 2016).

Auxin is the most important determinant of somatic embryogenesis for many species in tissue culture. Exogenous auxin promotes callus formation from cultured materials by inducing the production of endogenous precursors of ethylene synthesis, including 1-aminocyclopropane-1-carboxylic acid (Singla et al., 2007). 2,4-Dichlorophenoxyacetic acid (2,4-D), a synthetic auxin, is widely used in many species, especially cereal crops and medicinal plants. Gaj (2004) reported that in >65% of experiments, 2,4-D was used alone or combined with other hormones. The concentration of 2,4-D affects callus formation, and the optimal concentration varies for different species or tissues. The general principle is that a low concentration promotes embryonic callus formation, whereas a high concentration inhibits its formation. For most *Poaceae* spp. 2 mg/l of 2,4-D is optimal to induce embryonic callus formation (Wang et al., 2008; Çabuk and Özgen, 2016), and 5–10 μm 2,4-D is suggested for somatic embryos induction in many medicinal plants (Husain et al., 2010; Parveen and Shahzad, 2014). However, there is no need to add 2,4-D to medium after the embryonic callus develops into an embryoid and regenerates seedlings, suggesting that the effect of 2,4-D is promoted during embryogenic callus induction and inhibited during embryogenic callus development into a complete plant (Singla et al., 2007; Parveen and Shahzad, 2014). Additionally, different concentrations of auxins, such as indole-3-acetic acid (IAA) and α-naphthalene acetic acid, also play an important role in promoting the differentiation of adventitious roots in tissue culture (Nissen and Sutter, 1990; El-Sherif, 2018).

CK is the most widely used PGR in adventitious shoot induction and initiation of somatic embryogenesis in tissue culture. *De novo* shoot regeneration requires cell proliferation involving the activation of cell mitosis. CK affects competent cells in the shoot-regeneration process, leading to cell-mass generation and cell-fate transformation. CK can induce adventitious shoots alone and cooperates with auxin to reinforce proliferation in chosen cells (Cortleven et al., 2019). Skoog and Miller (1957) proposed that a high CK-to-auxin ratio stimulates shoot formation, whereas roots are formed when the ratio is low. To date, the golden hormone-regeneration pattern has been a guiding determinant of the fate of explants *in vitro*. In addition to inducing shoot regeneration, CK also initiated somatic embryogenesis. It was reported that MS medium containing 6-benzyladenine alone could induce high frequency of somatic embryo differentiation in *S. album* L. (Akhtar and Shahzad, 2019). Moreover, the effects of PGRs, such as abscisic acid (ABA) and gibberellin (GA), on plant regeneration have also been reported (Nishiwaki et al., 2000). The addition of GA to the medium promotes germination and differentiation of immature embryos, which inhibits somatic embryo development. Ge et al. (2016) reported that the maize transcription factor MYB138 promotes somatic embryogenesis by inhibiting GA signal transduction.

### Basal Culture Medium

Several types of culture media, including Murashige and Skoog (MS), N6, Woody Plant Medium (WPM), and B5, are used for callus induction and shoot differentiation and significantly influence plant regeneration in tissue culture (Table 1); however, different species or tissues may also require different basal medium. A previous study reported more prolific callus formation and higher shoot differentiation on MS medium than on B5 medium during plant regeneration from Easter lily (*Lilium longiflorum* L. cv. Ace) ovary tissues (Ramsay et al., 2003). However, N6 medium induced higher percentages of callus and green plants than did MS medium for rice (*O. sativa*; Sundararajan et al., 2017). For Indian siris (*Albizia lebbeck*), the WPM medium achieved the highest primary somatic embryoids development, whereas enhanced maturation of primary somatic embryoids occurred on MS medium (Saeed and Shahzad, 2015). During the conversion of somatic embryos into plantlets, a half strength MS medium performed better than other media in many medicinal plants (Sahai et al., 2010; Parveen and Shahzad, 2014; Saeed and Shahzad, 2015). Additionally, the carbon source is a vital component affecting plant regeneration in culture medium (Table 1). Sugar provides energy for the culture and represents the main regulator of the permeation environment, with glucose, sucrose, and maltose most often used in plant tissue culture. Small molecules of sugar can penetrate into living cells and dehydrate somatic embryos, thus promoting somatic embryo maturation (Kaviani, 2011). Moreover, a low sucrose concentration during somatic embryogenesis is advantageous to somatic embryo formation (Yaseen et al., 2013; Long et al., 2020). However, Malik et al. (2017) found that maltose resulted in maximal callusing and regeneration percentage as compared with other carbon sources for mature wheat embryos. Furthermore, compared with glucose and sucrose, maltose may more effectively inhibit the browning of plant cells. Other components, such as mannitol and metal ions, added to the culture medium can also affect the regeneration ability of explants (Simonović et al., 2021).

### Light/Dark Treatment

Under light conditions, phenolic compounds in explants will be oxidized by polyphenol oxidases, and the tissue will turn brown. The oxidation products can darken tissues and inhibit the activity of various proteins, with a potentially adverse effect on the formation of somatic embryos (Bhatia and Bera, 2015). Therefore, callus initiation, maintenance, and maturation require dark conditions in plant for many species. A previous report indicated that light reduces endogenous CK and auxin levels in plants by degrading auxins (Zenser et al., 2001). In this regard, darkness may help maintain a high auxin-to-CK ratio to support callus formation in explants. Additionally, dark conditions can lead to thinner cell walls and lower cell-wall deposits, thereby facilitating the entrance of PGRs into cells (Dai and Castillo, 2007). However, some studies have shown that light can promote callus formation by upregulating the expression of somatic embryogenesis marker genes, such as *WUS*, *BBM*, and leafy cotyledon 2 (*LEC2*; Siddique and Islam, 2015; Yu et al., 2019).

For shoot and root regeneration, a 16-/8-h photoperiod is generally required (Table 1). The frequency and speed of shoot initiation are higher under light conditions for maize regeneration (Li et al., 2002). A previous report showed that light might stimulate apical meristem differentiation by maintaining an optimal CK-to-auxin ratio, with low light intensity (~30–60 μmol m^−2^ s^−1^) preferable for shoot and root differentiation (Farhadi et al., 2017). Moreover, a recent study showed that light-emitting diodes (LEDs), which can regulate the level of photomorphogenic radiation necessary for plant morphogenesis, can be excellent substitutes for traditional cool-white fluorescent lamps (Bidabadi and Jain, 2020). Furthermore, LEDs are associated with cellular redox balance and involved in antioxidative metabolic activities during *in vitro* plant regeneration (Gupta and Karmakar, 2017).

## Molecular Mechanisms of Somatic Embryogenesis

**Figure 2:**
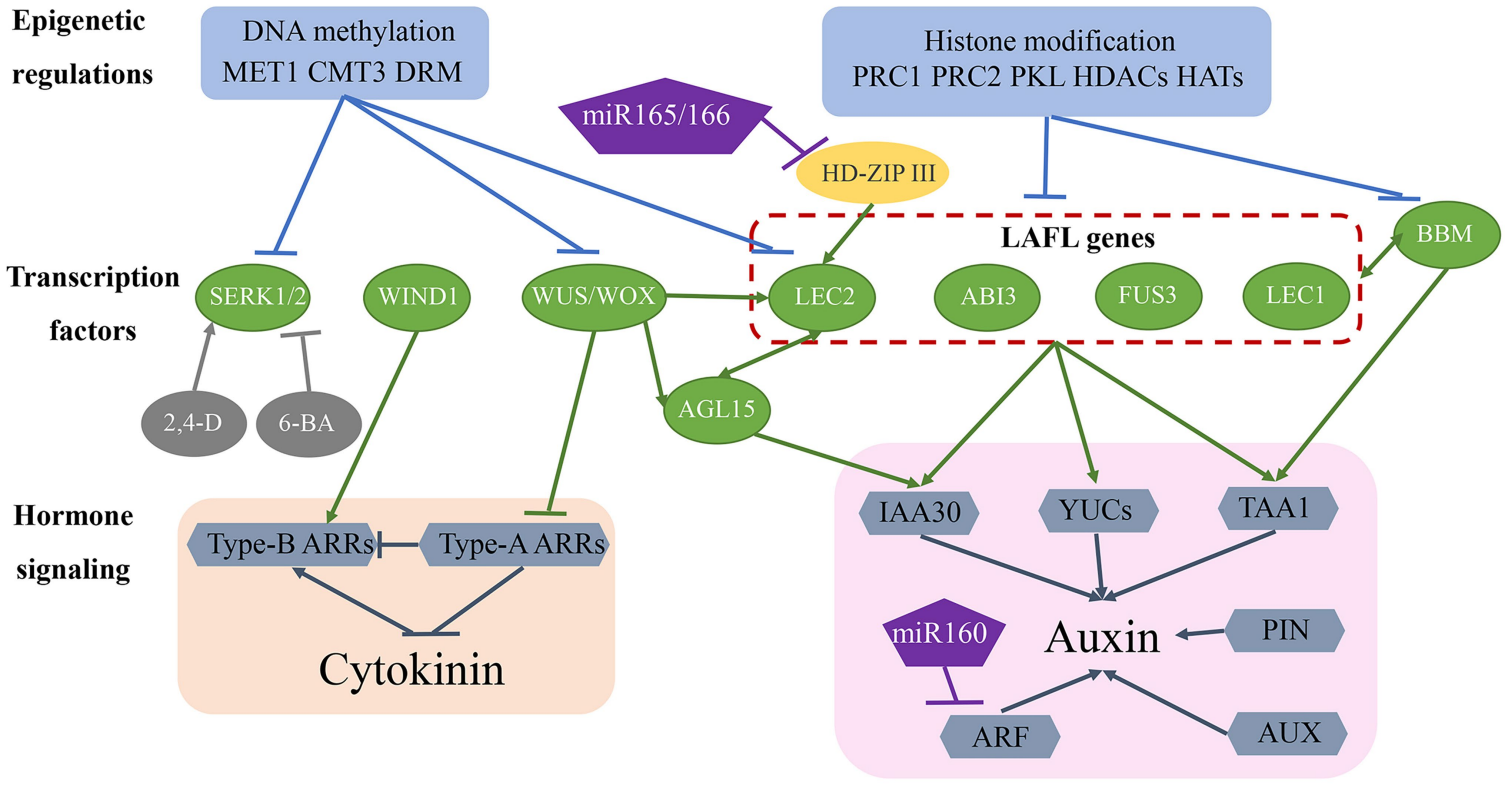
Molecular mechanisms of somatic embryogenesis. The somatic embryogenesis process is influenced by epigenetic regulation, transcription factors, and hormone signalling pathways. Epigenetic regulation, including DNA methylation and histone modifications, repress transcription factor access to gene-promoter regions, thereby inhibiting the expression of genes involved in somatic embryogenesis. Many transcription factors (green ovals) are involved in this regulatory network and also regulate each other and activate downstream auxin and CK signalling pathways. Additionally, miR-160 and miR-165/-166 are involved in regulating somatic embryogenesis.

Theoretically, somatic embryogenesis is a typical dedifferentiation process in which differentiated somatic cells are returned to the state of totipotent embryonic stem cells. Dedifferentiation is the basis of totipotency and regeneration in multicellular organisms. Recent research suggests that somatic embryogenesis is a complex process involving transcription factors, hormone signalling pathways, and epigenetic regulation (Figure 2).

### Transcription Factors

Several transcription factors have been identified as essential regulators of the somatic embryogenesis process (Figure 2; Table 1). Fusca 3 (*FUS3*), *LEC2*, and abscisic acid insensitive 3 (*ABI3*), encode plant-specific B3-domain-containing proteins that are members of the AFL subfamily of transcription factors (Parcy et al., 1994; Luerßen et al., 1998; Stone et al., 2001). These genes/proteins together with *LEC1*-encoded CCAAT-binding transcription factors harbouring a HAP3 subunit form LAFL complexes (Lotan et al., 1998; Lepiniec et al., 2018). Overexpression of each of these genes promotes the formation of somatic embryos or embryonic traits in somatic tissues in the absence of additional hormones (Xu and Huang, 2014). The expression of LAFL genes is regulated by epigenetic factors, hormone signalling, and other transcription factors, such as BBM (Salaün et al., 2021). *LEC2* and agamous-like 15 (*AGL15*) encode a MADS-box transcription factor that controls each of their respective expression in a regulatory feedback loop that also regulates the expression of the auxin-responsive protein gene *IAA30*, a primary factor in auxin signalling (Heck et al., 1995; Sato and Yamamoto, 2008). Additionally, LEC2 induces the expression of other auxin-related genes (*IAA1*, *IAA17*, and 1-aminocyclopropane-1-carboxylate synthase 4), as well as those encoding key enzymes involved in auxin biosynthesis, such as tryptophan aminotransferase of *Arabidopsis* 1 (*TAA1*) and *YUCCA* (*YUC*) genes (*YUC1*, *YUC2*, *YUC4*, and *YUC10*; Braybrook and Harada, 2008; Wójcikowska et al., 2013; Horstman et al., 2017). The BBM transcription factor upregulates the expression of LAFL genes and *AGL15* during the somatic embryogenesis process, and LAFL proteins regulate *BBM* expression (Horstman et al., 2017), with *BBM* overexpression promoting callus proliferation and formation of somatic embryos (Salaün et al., 2021).

The WUS homeobox-containing transcription factor is involved in regulating embryonic cell fate by inducing the vegetative-to-embryonic transition (Jha et al., 2020). Overexpression of WUS promotes somatic embryo production without requiring the addition of hormones in *Arabidopsis* (Zuo et al., 2002) and upregulates *LEC1*, *LEC2*, and *AGL15* expression during somatic embryogenesis (Jha et al., 2020; Figure 2; Table 1). Wus-related homeobox (*WOX*) genes encode similar sequences to the WUS homeodomain and a specific WUS box downstream of the homeodomain (Haecker et al., 2004). WOX proteins perform an essential role in early embryonic patterning (Salaün et al., 2021), and overexpression of WOX9 results in improved efficiency in somatic embryogenesis by increasing the levels of AGL15 and AGL8 (Tvorogova et al., 2019). Additionally, WOX5 is regarded as a marker of dedifferentiation based on its significant upregulation from the early stage of somatic embryogenesis (Orłowska and Kępczyńska, 2018).

Wound-induced differentiation 1 (*WIND1*), which encodes another APETALA2/ethylene-responsive element-binding factor transcription factor, induces the acquisition of regeneration competency (Iwase et al., 2011); however, it is not directly involved in promoting somatic embryo formation, although it does play a role in the induction of callus in the indirect somatic embryogenesis pathway. Similar to WUS, WIND1 acts upstream of LEC2 during regeneration (Iwase et al., 2015). Compared with LAFL proteins, WUS and WIND1 induce somatic embryogenesis through a different hormone pathway (Figure 2; Table 1) and are mainly involved in CK-specific responses rather than auxin biosynthesis and signal transduction (Horstman et al., 2017). Specifically, WUS represses negative regulators [type-A *Arabidopsis* response regulator (*ARR*) genes] of CK response, whereas WIND1 stimulates the expression of positive regulators (type-B *ARR* genes) of CK response (Leibfried et al., 2005; Iwase et al., 2011).

Somatic embryogenesis receptor-like kinase (*SERK*) belongs to the RLK gene family, and as the first key gene screened in a carrot hypocotyl regeneration study, it regulates the transition from somatic cells to embryonic cells (Schmidt et al., 1997). Studies show that single cells expressing *SERK* can develop into regenerative somatic embryos, with regenerative somatic cells and zygotic embryos demonstrating the same signal transduction pathway. *SERK* genes were subsequently cloned from *Arabidopsis* (Hecht et al., 2001), rice (Hu et al., 2005), wheat (Singla et al., 2008), maize (Zhang et al., 2011), and other plants and showed higher expression levels in the embryogenic callus and maturation stage than in the non-embryogenic callus (Gulzar et al., 2020). In maize, *Zm*SERK1 and *Zm*SERK2 exhibit redundant functions in the initiation of embryonic cell formation and division and are regulated by auxin and CK (Zhang et al., 2011). Additionally, 2,4-D enhances *Zm*SERK1 and *Zm*SERK2 levels, which promote somatic embryogenesis, whereas the CK 6-benzyladenine reduces their respective expression, thereby inhibiting somatic embryogenesis (Zhang et al., 2011; Méndez-Hernández et al., 2019; Figure 2; Table 1).

Several other transcription factors are also critical for regulating somatic embryogenesis. PGA37/MYB118 and MYB115 promote somatic embryo formation by positively regulating the expression of *lec1* (Wang et al., 2009). Additionally, LEC1-like, the most closely related subunit of LEC1, plays an important role in embryogenesis (Kwong et al., 2003). Furthermore, a double mutant of the genes viviparous1/ABI3-like 1 (*VAL*)*1* and *VAL2* exhibited embryo-like proliferations, suggesting that VAL1 and VAL2 negatively regulate somatic embryogenesis (Suzuki et al., 2006).

### Hormone Signalling Pathway

Plant hormones, especially auxins and CKs, are key factors in the somatic embryogenesis pathway. Therefore, genes associated with hormone signalling pathways are likely to play an important role in that process (Figure 2; Table 1). The LAFL protein complex upregulates the expression of auxin-biosynthesis-related genes (*TAA1* and *YUC* genes) and the auxin signalling gene *IAA30*, and WUS and WIND1 negatively and positively regulate type-A *ARR* and type-B *ARR* genes corresponding to CK responses. Additionally, polar auxin transport induces concentration gradients maximal necessary for plant development. Pin-formed (PIN) and AUX proteins achieve differential distributions by controlling auxin efflux and influx, respectively (Petrásek and Friml, 2009). Moreover, differential expression of *AUX/IAA* genes and auxin response factors (ARFs), the core components of the auxin signalling pathway, is related to induction of somatic embryogenesis (Quintana-Escobar et al., 2019; Wójcik et al., 2020). Furthermore, microRNA (miR)-165/-166 and miR-160 may contribute to auxin-related induction of somatic embryogenesis by targeting the HD-ZIP III family genes phabulosa/phavoluta (*PHB/PHV*), positive regulators of *LEC2* expression, and ARF genes (*ARF10*, *ARF16*, and *ARF17*), respectively (Wójcik et al., 2017; Jin et al., 2020).

### Epigenetic Regulation

Epigenetic regulation is key to maintaining somatic cell identity by suppressing the expression of embryo-specific genes (Figure 2; Table 1). DNA methylation and histone modification play an important role in regulating gene expression and determining cell fate (Méndez-Hernández et al., 2019). During callus formation, DNA methyltransferase activity regulates gene transcription. A previous study showed that mutation in methyltransferase 1 (*MET1*) results in decreased CG methylation and dysregulated expression of the auxin efflux carrier PIN1 engaged in polar auxin transport during somatic embryogenesis (Wójcikowska et al., 2020). Decreased methylation has been reported in *SERK*, *LEC2*, and *WUS* in the embryogenic callus (Karim et al., 2018). Additionally, studies revealed relatively lower levels of DNA methylation at CG, CHG, and CHH sequence contexts in association with MET1, chromomethylase 3 (CMT3), and domains rearranged methyltransferase 2 (DRM2) activities related to somatic embryogenesis and regeneration ability (Karim et al., 2018; Wójcikowska et al., 2020).

In addition to DNA methylation, histone modifications, including methylation, acetylation, and ubiquitination, also play an important role in regulating somatic embryogenesis. Polycomb repressive complex (PRC)1 and PRC2 are required to establish and maintain stable epigenetic suppression in response to developmental or environmental signals (Mozgova and Hennig, 2015; Figure 2; Table 1). PRC2 exhibits histone 3 lysine 27 trimethylation (H3K27me3) activity, and *PRC2* mutation results in incomplete transition from embryo to seedling, disorderly cell division in seedlings, and formation of callus with embryo traits (Xu and Huang, 2014). PRC1 recognizes H3K27me3 alterations and promotes chromatin compaction *via* histone H2A lysine ubiquitination (Salaün et al., 2021). A recent study showed that PRC1 and PRC2 repress the expression of embryo-specific genes, including *LAFL*, *AGL15*, *WOX5*, *BBM*, and *PIN1* (Duarte-Aké et al., 2019). Additionally, pickle (PKL), a member of the chromodomain helicase DNA-binding protein 3 family of chromatin ATPase remodelers, is another epigenetic factor that plays a key role in preventing somatic cells from producing embryonic traits (Ogas et al., 1999). Similar to PCR1 and PCR2, PKL represses the expression of embryonic genes, including LAFL genes, by promoting H3K27me3 alterations (Dean Rider et al., 2003; Aichinger et al., 2009; Figure 2). Furthermore, histone acetylation regulated by histone acetyltransferases (HATs) and histone deacetylases (HDACs) plays a critical role in somatic embryogenesis (Tanaka et al., 2008; Figure 2). Trichostatin A, an HDAC inhibitor, upregulates the expression of genes related to embryogenesis, including *LEC1*, *FUS3*, and *ABI3* (Tanaka et al., 2008).

## Molecular Mechanisms of *de novo* Root Organogenesis

### Formation of Pluripotent Callus

**Figure 3:**
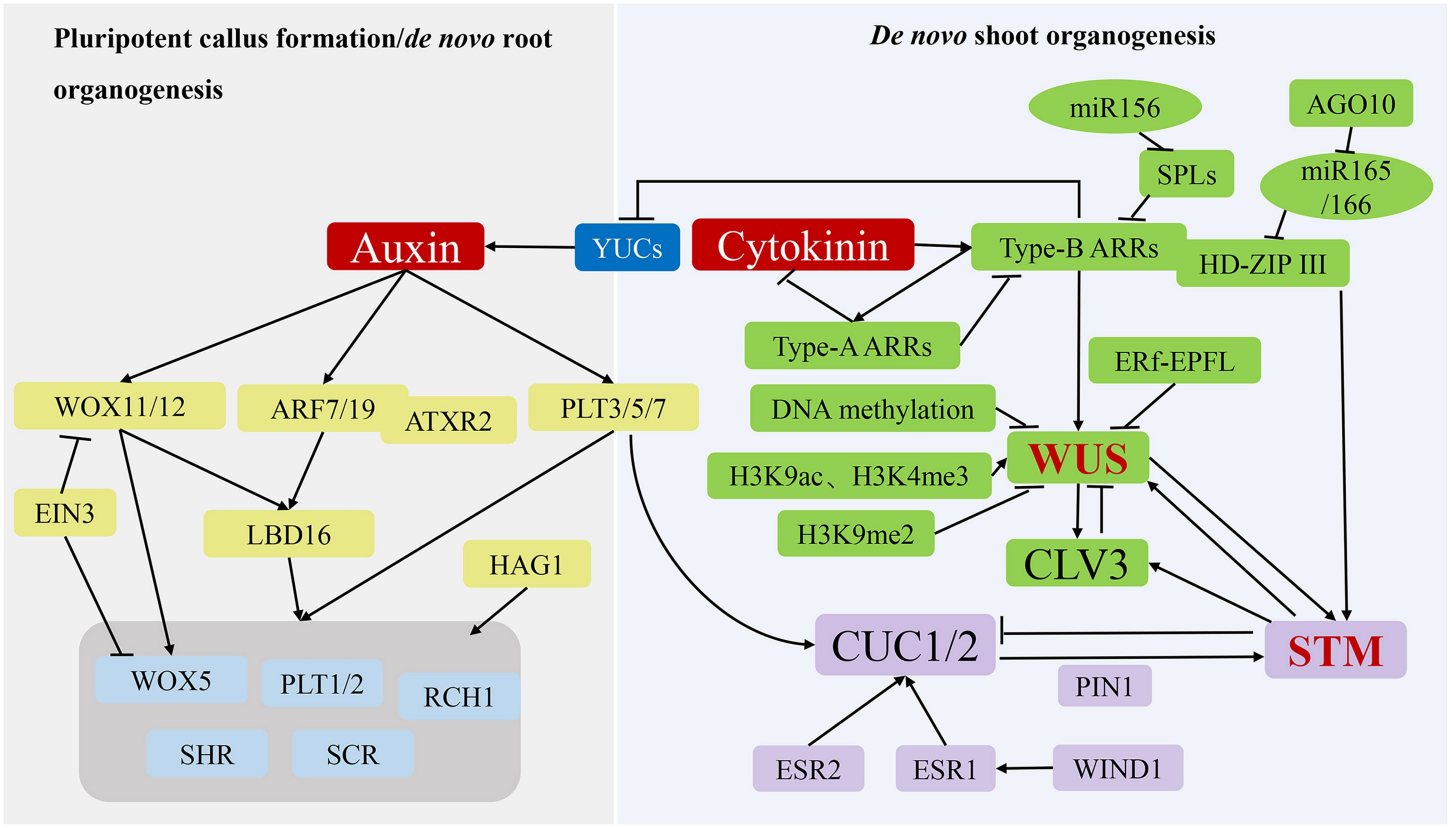
Molecular mechanisms of *de novo* organogenesis in tissue culture. During the process of pluripotent callus formation or *de novo* root organogenesis (left panel), YUC-mediated auxin acts as a key regulator to activate *WOX11/12*, *ARF7/19* and *PLT3/5/7* expression, after which their translated products directly or indirectly promote the expression of genes, including *WOX5*, *PLT1/2*, *SHR*, *SCR*, and *RCH1*, to induce pluripotent callus or root apical meristem formation. During the process of *de novo* shoot organogenesis (right panel), two pathways (WUS-CLV3 and STM-CUC) establish negative-feedback loops and play critical regulatory roles. The WUS-CLV3 pathway is mainly regulated by DNA methylation, histone modification, and hormone signalling. CK activates the expression of type-B *ARRs* to stimulate *WUS* expression, whereas type-B ARRs repress YUC-mediated auxin biosynthesis. In the STM-CUC pathway, *STM* expression is promoted by CUC1 and CUC2, both of which have their expression upregulated by PLT3/5/7, ESR1, ESR2, WIND1, and PIN1. Moreover, WUS and STM interact directly to activate *CLV3* expression, suggesting that the two pathways converge and coordinate to control shoot regeneration.

Pluripotent callus formation is initiated by the division of pericycle cells in the xylem pole in a process similar to lateral root initiation (Atta et al., 2009), with molecular factors participating in lateral root initiation also involved in pluripotent callus formation. During this process, some root meristem marker genes, including *WOX5*, scarecrow (*SCR*), short root (*SHR*), plethora (*PLT*)*1*, *PLT2*, and root clavata-homolog 1 (*RCH1*), are significantly upregulated (Atta et al., 2009; Figure 3; Table 1). *WOX5*, *SCR*, *PLT1*, and *PLT2* are transcriptionally activated by HAT of the GNAT/MYST superfamily 1, which binds directly to their respective promoters to initiate acetylation (Kim et al., 2018). Additionally, the rapid induction of *PLT3*, *PLT5*, and *PLT7* expression by auxin results in transcriptional regulation of *PLT1* and *PLT2* (Kareem et al., 2015). Moreover, WOX11 promotes pluripotency acquisition by activating the expression of lateral organ boundaries domain 16 (*LBD16*), which is activated *via* ARFs and promotes the expression of *WOX5*, *PLT1*, and *PLT2* (Xu and Hu, 2020).

### Molecular Mechanisms of Root Apical Meristem Formation

In tissue culture, *de novo* root organogenesis is induced by transferring pluripotent callus to root induction medium rich in auxin. In past years, the analysis of transcriptome, epigenome, and cell lineage in pluripotent callus has revealed that the formation of pluripotent callus and *de novo* root organogenesis share similar genetic pathways (Liu et al., 2014; Xu and Huang, 2014; Figure 3; Table 1). The *de novo* root organogenesis process can be divided into two steps: the transition of competent cells to root founder cells, which is marked by *WOX11*, and the switch of root founder cells to root primordium cells, which is marked by *WOX5* (Liu et al., 2014). Inhibition of polar auxin transportation blocks the rooting process, suggesting that auxin is the key hormone that regulates *de novo* root organogenesis (Greenwood et al., 2001). Suppression of *YUC* genes (*YUC1, 2, 4, 5, 6, 8*, and *9*) mediating auxin biogenesis inhibits the expression of *WOX11* and prevents the fate transition of competent cells (Chen et al., 2016; Pan et al., 2019).

In the first step, auxin directly activates the expression of WOX11 and its homolog *WOX12* (Sang et al., 2018; Figure 3; Table 1). During the next step, *WOX11/12* promotes the expression of *WOX5* and *LBD16* responsible for activating the expression of *WOX5*, *PLT1*, and *PLT2* (Sang et al., 2018). It was found that the transcription factor ethylene insensitive 3 (*EIN3*) strongly decreased the *de novo* root organogenesis rate by suppressing the transcription of *WOX11* and *WOX5* (Li et al., 2021; Figure 3; Table 1), and older explants showed increased *EIN3* activity, which is in accord with the observation that younger organs possess a higher regeneration ability (Li et al., 2021). As mentioned above, auxin also induces *PLT3*, *PLT5*, and *PLT7* expression, which subsequently regulate downstream root meristem marker genes. In addition to *WOX11/12* and *PLTs* genes, the auxin response factors *ARF7* and *ARF19* target and activate the expression of *LBD16* (Okushima et al., 2007). The Arabidopsis trithoprax-related 2 (ATXR2) protein can physically interact with ARF7 and ARF19. The complex catalyses H3K36me3 deposition at the promoter of *LBD16* to promote its expression in the root regeneration process (Lee et al., 2018).

### Molecular Mechanisms of *de novo* Shoot Organogenesis

After culturing on SIM rich in CK, the pluripotent callus continues to divide under CK-mediated actions, and cell populations gradually generate for subsequent differentiation, signifying the construction of the stem cell niche (Ikeuchi et al., 2019). Shoot stem cell homeostasis is maintained by two regulatory pathways: WUS-clavata 3 (CLV3) and shoot meristemless (STM)-cup-shaped cotyledon (CUC; Figure 3; Table 1). As the determining factor in the early stage of stem cell niche construction, *WUS* expression begins 2 to 3 days after SIM culture (Zhang et al., 2017), with initial expression of *WUS* marking the establishment of shoot-progenitor cells and representing the most critical molecular event in *de novo* shoot organogenesis. The regeneration ability of the *WUS* mutant is completely lost, whereas *WUS* overexpression results in ectopic formation of shoots, indicating that *WUS* is necessary for *de novo* shoot regeneration (Gordon et al., 2007). WUS promotes the expression of *CLV3*, which encodes a signal peptide, whereas CLV3 inhibits *WUS* expression in a negative-feedback loop that plays a key role in maintaining the stem cell population (Han et al., 2020). Similarly, STM is expressed throughout the shoot meristem and represses the expression of *CUC1* and *CUC2*, whereas CUC1 and CUC2 activate *STM* expression to maintain the shoot meristem (Balkunde et al., 2017).

The WUS-CLV3 pathway is regulated by DNA methylation, histone modification, and hormone signalling (Figure 3). Mutations of *MET1*, *CMT3*, *DRM1*, and *DRM2* result in loss or reduction in DNA methylation in the regulatory regions of the *WUS* promoter, which enhances *WUS* expression and the shoot-regeneration rate (Sugimoto et al., 2019). *WUS* gene promotes both somatic embryogenesis and *de novo* organogenesis (Figures 2, 3) so that the lower levels of DNA methylation at CG, CHG, and CHH sequence contexts in association with MET1, CMT3, DRM1, and DRM2 activities are beneficial for two pathways. In somatic embryogenesis pathway, H3K27me3 alterations prevent somatic cells from producing embryonic traits by repressing the expression of *WUS* gene. However, *de novo* shoot regeneration involves different histone modification sites at WUS. The abundance of markers of histone 3 lysine 9 acetylation (H3K9ac) and histone 3 lysine 4 trimethylation (H3K4me3) at WUS sites increases, whereas the abundance of inhibitory markers histone 3 lysine 9 di-methylation (H3K9me2) decreases during shoot regeneration (Li et al., 2011). By contrast, kryptonite, an H3K9 methyltransferase, and Jumanji-domain-containing 14, an H3K4 demethylase, are responsible for repressing *WUS* transcription, which decreases shoot production. However, HAC1, a HAT, and lysine-specific demethylase 1-like 3, an H3K4 demethylase, activated *WUS* transcription, which increased shoot production (Ishihara et al., 2019).

Additionally, the auxin and CK signalling pathways affect *WUS* expression. As transcriptional activators of CK signalling, type-B ARRs (ARR1, ARR2, ARR10, and ARR12) directly activate *WUS* expression following binding to its promoter (Zhang et al., 2017), while also suppressing YUC-mediated auxin accumulation to further promote *WUS* expression (Meng et al., 2017). Type-A ARRs (ARR5, ARR6, ARR7, and ARR15), as negative regulators of CK signalling, are directly regulated by type-B ARRs and interfere with the function of type-B ARRs, thereby creating a negative-feedback loop (Sugimoto et al., 2019). Furthermore, targeting of squamosa promoter binding protein-like (*SPL*) mRNA by miR-156 decreases regulation of the activities of type-B ARRs in an age-dependent manner (Zhang et al., 2015). In young explants, miR156 levels are elevated relative to those in adult explants and repress *SPL* expression, thus increasing type-B ARR activity and shoot-regeneration ability. Moreover, miR-165/-166 inhibits shoot regeneration by splicing and reducing the translation of mRNAs encoding proteins harbouring an HD-ZIP III domain, including PHB, PHV, and REVOLUTA (Shin et al., 2020). Argonaute 10 inhibits shoot regeneration by suppressing miR-165/-166 activity. Another study found that type-B ARRs interact with HD-ZIP III proteins to form transcription complexes that specifically activate *WUS* expression during the early stage of shoot regeneration (Zhang et al., 2017), and a recent study demonstrated that accurate spatial expression of *WUS* and *CLV3* influences their function (Zhang et al., 2021). Specifically, a signalling pathway comprising ERECTA family receptors and epidermal-pattern factor-like ligands inhibits the expression of *WUS* and *CLV3* in the periphery of the shoot apical meristem, confining them to the centre. These findings demonstrate that *WUS* expression is determined by multiple regulators in a complicated molecular network.

In the STM-CUC pathway, the negative-feedback loop between *STM* and *CUC* plays a critical role in regulating *de novo* shoot organogenesis (Figure 3; Table 1). CUC proteins are essential in establishing the shoot promeristem (Aida et al., 1999). Polar localisation of PIN1 induced by CUC determines the location of shoot progenitors, with the polarized upregulation of PIN promoting *STM* expression in the promeristem (Gordon et al., 2007) Additionally, PLT3, PLT5, and PLT7 upregulate *CUC1* and *CUC2* expression during shoot regeneration, with these PLT proteins controlling shoot regeneration *via* a two-step mechanism that first establishes competence by activating *PLT1* and *PLT2* expression during pluripotent callus formation. Moreover, PLTs regulate CUCs to accomplish regeneration (Kareem et al., 2015). In addition to PLTs, enhancer of shoot regeneration (ESR)1 and ESR2 act as upstream regulators of *CUC* genes during *de novo* shoot organogenesis by activating *CUC1* expression by directly binding to its promoter (Ikeda et al., 2006; Matsuo et al., 2011). Notably, *ESR1* expression is regulated by WIND1, which connects wound signalling to shoot regeneration (Iwase et al., 2017).

Both the WUS-CLV3 and STM-CUC pathways are essential for stem cell development during *de novo* shoot organogenesis. A recent study reported that the two pathways converge and coordinate through direct interaction between the WUS and STM proteins (Su et al., 2020; Figure 3; Table 1). Specifically, STM directly activates *CLV3* expression by binding to its promoter at a different site from that of WUS. Additionally, WUS–STM interactions enhance WUS binding to the *CLV3* promoter and activation of *CLV3* transcription, suggesting that *CLV3* is simultaneously regulated by *WUS*, *STM*, and their complex (Su et al., 2020; du and Homeostasis, 2020). Furthermore, STM activity is regulated by WUS activity in the shoot meristem (Lenhard et al., 2002; Su et al., 2020).

## Discussion

### Application and Challenge

Plant-regeneration techniques in tissue culture have been used in many fields, including gene-function research, transgenic breeding, and rapid micropropagation. In gene-function research, multiple methods, including overexpression, gene knockout, and genome editing, rely on genetic transformation in plants. The embryogenic callus is the most widely used genetic transformation receptor in most species. For example, CRISPR-Cas9 promoter editing of maize *Arabidopsis CLV3-LIKE* genes enhanced grain-yield-related traits (Liu et al., 2021). However, only a few plant lines can establish an efficient transgenic system. Genotype has become the inhibitory factor in genetic transformation and gene-function verification. Therefore, understanding the mechanisms associated with embryogenic callus induction and plant regeneration can facilitate gene-function validation and research.

In addition to gene-function research, transgenic plant breeding is also based on genetic transformation. Compared with traditional breeding, transgenic technology can break the reproductive isolation between species, realize the precision improvement of certain genes, estimate offspring traits, and offer the advantages of accurate targeting and shorter breeding cycles (Gepts, 2002). However, genotype limitations to genetic transformation inhibit the development of transgenic plant breeding. In the case of maize transgenic breeding, most backbone lines used for commercial production are recalcitrant to transformation, resulting in the desirable gene needing to first be introduced into a few good transgenic receptors, followed by import of the desirable gene fragment into the target inbred line through successive backcrosses. Therefore, conventional maize breeding systems must undergo genetic transformation, successive self-pollinations, and backcrosses that require at least 3 to 6 years and greatly extends the maize transgenic breeding cycle. Hence, analysing the mechanism of plant regeneration can create more transgenic receptors, address genotype-specific limitations, and further accelerate the transgenic breeding process.

Micropropagation is among the most important plant tissue culture techniques because of its ability to rapidly multiply a selected plant with a minimal number of starting materials. Compared with conventional propagation by seeds or vegetative methods, micropropagation enables large-scale propagation of multiple plants, resulting in its wide use in research and commerce. Moreover, micropropagation is an efficient technology for preserving gene pools and genetic diversity in plants (Chokheli et al., 2020). Many endangered or rare species have been successfully propagated using micropropagation, including *Artemisia hololeuca* and *Hyssopus angustifolius* (Zayova et al., 2018; Chokheli et al., 2020). Furthermore, many high-demand medicinal plants have been mass-developed using micropropagation (Moraes et al., 2021). Efficient regeneration depends on an appropriate micropropagation protocol, including explant types, medium compositions, and culture conditions (Singh, 2015). Therefore, understanding the plant-regeneration mechanism promotes the use of effective protocols for plant micropropagation.

## Conclusion and Future Perspectives

In this review, we discussed how regeneration happens through two different pathways (somatic embryogenesis and *de novo* organogenesis in tissue culture) and the environmental factors and molecular mechanisms affecting these two pathways. This information offers a reference for scientific research and technology development in this field.

Despite the extent of research and the remarkable advances made as a result, the mechanisms that regulate plant regeneration require further elucidation. Plant regeneration *in vitro* is a complex developmental process, with only part of this process currently understood and requiring additional study for a comprehensive and integral understanding. First, although the regulatory network involved in plant regeneration has been initially determined, how these players and signalling molecules coordinate the different stages of regeneration remains unclear. Second, although we understand that plant regeneration is regulated by complex networks of gene regulation and influenced by external environmental stimulation, the interaction between external and internal signals to achieve the dynamic balance of growth and development requires further investigation. Specifically, it is unclear how external signals selectively activate internal plant regulators. Therefore, future studies on regeneration mechanisms should explore the interaction between external environmental factors and internal signalling networks. In tissue culture, the traditional way to improve plant regeneration is to change external environmental factors; therefore, combining an understanding of molecular mechanisms with traditional methods to achieve targeted plant regeneration should be a focus of future research. Third, the factors that control plant regeneration have mainly been outlined in *Arabidopsis*; however, whether other plants exhibit the same molecular mechanisms remains unverified. Rapid micropropagation and genetic transformation of most important crops and medicinal plants remain difficult; therefore, a future developmental direction for plant-regeneration research might involve applying theoretical concepts of plant-regeneration mechanisms to agricultural practice in order to help establish efficient regeneration systems and promote the industrialisation of agricultural biotechnology. Finally, the computer modelling, based on integral understanding, might be a promising research direction in plant tissue culture. In future, we just input the genotype of the species, then select the explant sources and desired regeneration pathway, the computer may automatically design the culture conditions we need, such as the composition of the culture medium and the amount of PGRs. Or we tell computer the genetic information and environmental conditions used for a certain species, it might simulate the entire culture process and the expected outcomes. That would greatly accelerate the research process of plant tissue culture.

## Author Contributions

YL wrote the initial manuscript. YY prepared these figures. YS and GP reviewed the manuscript and made significant editorial contributions. All authors contributed to the article and approved the submitted version.

## Funding

This work was supported by the Fundamental Research Funds of China West Normal University (412906).

## Conflict of Interest

The authors declare that the research was conducted in the absence of any commercial or financial relationships that could be construed as a potential conflict of interest.

## Publisher’s Note

All claims expressed in this article are solely those of the authors and do not necessarily represent those of their affiliated organizations, or those of the publisher, the editors and the reviewers. Any product that may be evaluated in this article, or claim that may be made by its manufacturer, is not guaranteed or endorsed by the publisher.
